# Buccal fat pad removal to improve facial aesthetics: an established technique?

**DOI:** 10.4317/medoral.22449

**Published:** 2018-06-21

**Authors:** Lucas-Borin Moura, José-Rodolfo Spin, Rubens Spin-Neto, Valfrido-Antonio Pereira-Filho

**Affiliations:** 1 DDS, PhD Student - Department of Diagnosis and Surgery, Division of Oral and Maxillofacial Surgery, Dental School at Araraquara – UNESP – São Paulo State University, Brazil; 2 DDS, MS Student - Department of Diagnosis and Surgery, Division of Oral and Maxillofacial Surgery, Dental School at Araraquara – UNESP – São Paulo State University, Brazil; 3 DDS, MS, PhD - Department of Dentistry and Oral Health, Section of Oral Radiology, Faculty of Health, Aarhus University, Denmark; 4 DDS, MS, PhD - Department of Diagnosis and Surgery, Division of Oral and Maxillofacial Surgery, Dental School at Araraquara – UNESP – São Paulo State University, Brazil

## Abstract

**Background:**

Buccal fat pad (BFP) is a singular structure between the facial muscles. Its removal may enhance the zygomatic prominences resulting in an inverted triangle of beauty. Objective: The aim of this study was to perform a systematic review of literature about BFP removal for facial aesthetic improvement. In order to answer the following research question: What are the indications, complication types and rates, surgical techniques and outcomes of the technique?

**Material and Methods:**

The initial search in Pubmed, Scopus, and Cochrane databases recognized 220 articles. The final review included eight of them. None of the included studies were clinical trials.

**Results:**

BPF removal was performed by intraoral incision or associated with the face lift procedure. In 71 patients submitted to the procedure and evaluated about complications, only 8.45% presented minor complications. Parotid duct and facial nerve injuries were not found. No study evaluated facial aging and long-term effects, therefore the harmless effect of the procedure to those features is not clear.

**Conclusions:**

Although it is not a novel procedure, there is a lack of information about long-term outcomes. Thus, controlled clinical studies should be performed to achieve adequate clinical evidence of those aspects.

** Key words:**Buccal fat pad, facial sculpting, cheek surgery, buccal lipectomy.

## Introduction

The buccal fat pad (BFP) is a rounded biconvex adipose structure limited by a thin capsule. It is located in the middle third of the cheek and composed by three lobes. The anterior lobe protrudes in front of the anterior border of the masseter muscle. The intermediate one extends between the masseter and buccinators muscles. And the posterior lobe continues between temporal masticatory space (1). Therefore, the BFP has intimate relationship with the masticatory system, facial nerve, and parotid duct (1,2).

The BFP was firstly described by Heister as a glandular tissue in 1732. However, in 1802 Bichat popularized and defined the structure as fat tissue (1-3). This specialized fat has function of smooth gliding between muscles to enhance the intermuscular motion. In theory, this function occurs especially during the infant suckling, and explain the large BFP volume in infants and small one in adults (3-5).

Anatomically, the lower face contour is composed by four elements: BFP, masseter muscle, mandibular bone, and the subcutaneous fat. Thus, the BFP has an important role in the facial aesthetics. If the buccal extension is excessive, patients may complain of rounded face, excessive cheeks or “baby faces” (1,6-8). Therefore, the BFP removal or “partial buccal lipectomy” is presented as a technique to sculpt the facial angles and enhance aesthetics (6-8).

There are two methods to perform BFP removal, through intraoral approach or by facial approach during the facelift procedure. According to the literature, the safest method is to perform an intraoral incision (5,9). Usually, the intraoral BPF removal is performed under local anesthesia and the incision is performed in the maxillary gingivobuccal sulcus (3,8-10) or in buccal mucosa at bite level (7). After incision, the buccal muscle is dissected and the BFP is exposed. An external pressure is applied over the skin to manipulate the BFP into the incision, and without excessive traction the exposed portion is clamped and excised. An absorbable suture is used to close the wound (8-10). The potential complications include: hematoma, trismus, infection, facial nerve impairment, parotid duct injury, over resection, induration, and asymmetry (3,6,10).

Some authors consider the BFP removal a safe procedure to enhances the facial aesthetics (7). However, to the best of our knowledge, nowadays the procedure is disseminated as routine especially in Brazil. Therefore, this study aims to perform a systematic review of the literature about BFP removal to improve facial aesthetics, regarding to the immediate effects, outcomes, and complication rate.

## Material and Methods

This systematic review was directed in accordance with PRISMA (Preferred Reporting Items for Systematic Reviews and Meta-Analyses) (11).

-Criteria for considering studies for this review

Studies in which the methodology and/or results included information regarding the surgical excision of the BFP to improve facial aesthetics qualified for inclusion. Also, eligibility criteria include: studies published in English; without time limitations; studies of human beings; prospective, clinical studies and case series/report. If the study approached the topic of buccal fat pad excision as part of grafting or other non-aesthetic procedures, it was excluded.

-Search strategy for study identification 

-Electronic search

The MEDLINE (Medical Literature Analysis and Retrieval System Online, via PubMed), Elsevier (via SCOPUS) and Cochrane Library databases were searched for studies regarding the surgical excision of the buccal fat pad aiming at aesthetical optimization. The search strategy was restricted to English language publications using the following sequence of terms adapted to each database: (*(buccal fat pad) OR (bichat) OR (corpus adiposium buccae) OR (cheek fat) AND ((buccal lipectomy) OR (lipectomy) OR (removal) OR (bichatectomy)*).

Systematic reviews and reviews were immediately excluded. A secondary search was conducted based in the titles, abstracts, and keywords of the analyzed studies, to check if they would fit to the topic. Eventually, the full texts were read and the final study selection was conducted.

-Unpublished data and hand-search

Unpublished data were sought by searching a database listing unpublished studies (OpenGray - www.opengrey.eu). A manual search was additionally conducted based on the reference lists of the selected papers and of other previous reviews. Electronic databases of the following journals, which were considered important to this review, were searched: International Journal of Oral and Maxillofacial Surgery, Journal of Oral and Maxillofacial Surgery, Journal of Craniomaxillofacial Surgery, Journal of Craniofacial Surgery, British Journal of Oral and Maxillofacial Surgery, Craniomaxillofacial Trauma and Reconstruction, Journal of Periodontology, Journal of Clinical Periodontology, Clinical Oral Investigations, Dentomaxillofacial Radiology, Medicina Oral Patología y Cirugia Bucal, Oral Surgery, Oral Medicine, Oral Pathology and Oral Radiology, Clinical Oral Implants and Related Research and Journal of Oral and Maxillofacial Implants, Journal of Plastic and Reconstructive Surgery, Journal of Aesthetic Plastic Surgery, Journal of Clinical Otorhinolaryngology, Head & Neck Journal, Annals of Plastic Surgery, Periodontology 2000, Clinical Periodontology.

-Study selection and data extraction

Titles, abstracts, and full texts of the articles were independently screened by three researches (LBM, JRS and RSN). When there was a disagreement, the reviewers discussed the study and reached a consensus. Also, those researchers conducted data extraction and the validity assessment of the studies that met the inclusion criteria. Data was extracted focusing on surgical technique (preoperative image exam, incision site, anesthesia regimen, and associated procedures) and complications (type and rate) associated with buccal fat pad removal for facial aesthetics improvement.

-Quality evaluation

Quality evaluation was performed using PRISMA statement (11) criteria to evaluate the strength of the scientific evidence present. All included studies were classified according to the potential risk of bias: random sample selection; definition of inclusion and/or exclusion criteria, follow-up loss report, validated measurements obtained, and presence of statistical analysis. Studies classified as low risk of bias had all criteria presented; moderate risk of bias had absence of one of the criteria; and high risk of bias had absence of two or more criteria.

## Results

**Figure 1:**
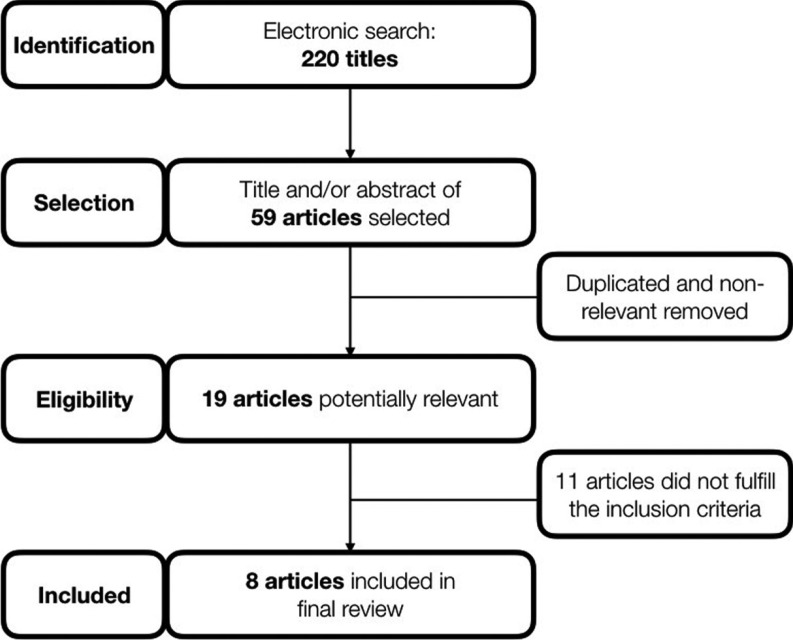
Flowchart of systematic review process.

The final electronic search was performed in January 2018, and identified 220 articles (180 Pubmed, 39 Scopus, 1 Cochrane Library, none OpenGray). Twenty were selected as potential relevant studies after title and/or abstract reading, one full-text of those could not be obtained (13). Full-text of 19 studies were read, but 11 did not fulfill one or more inclusion criteria. Eight articles were included in the final review (2-4,6-7,10,14-15). Figure 1 shows the flowchart of eligibility and evaluation process.

None of included articles were retrospective or prospective clinical trials, four were case series (3,6-7,10), three case reports (4,14-15), and one experience report based on almost two-hundred surgical cases (2). About quality evaluation, all articles achieve a high risk of bias ( Table 1).

**Table 1:**
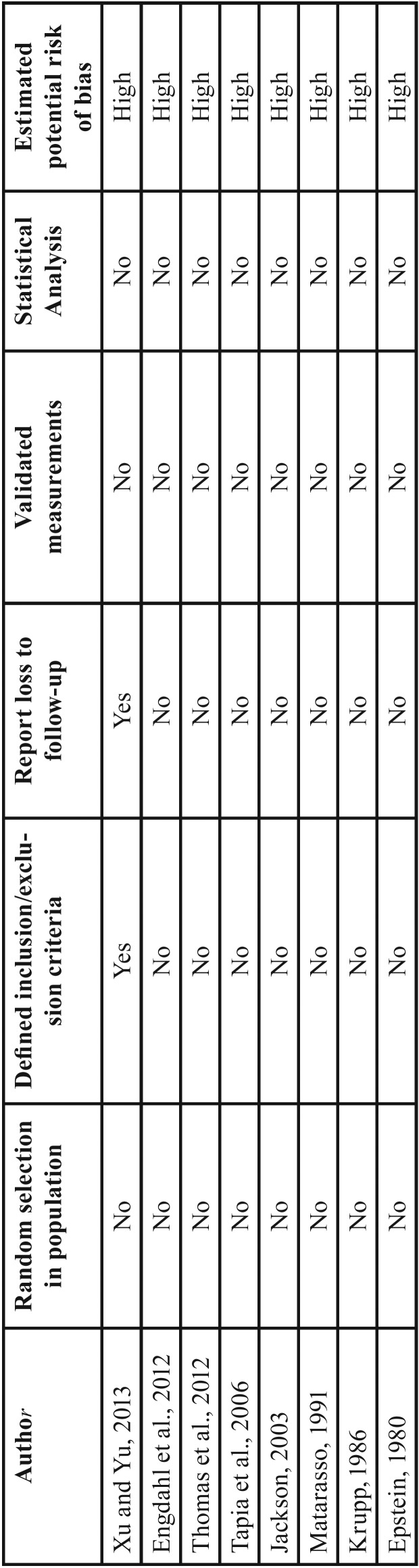
Quality assessment of the studies.

 Table 2 shows data extracted of the included articles. Regarding to surgical technique there was two approaches to the BFP: through intraoral incision or by facial approach. Rhytidectomy (facial approach) was performed just in one study (2), which describes the BFP removal associated to face lift procedure. The intraoral incision (3-4,6-7,10,14-15) was performed in two regions: at bite level (7) or at maxillary gingivobuccal sulcus (3,6,10).

Five studies reported patients age and gender (3-4,7,13-14). The age ranged from 18 to 60 years, suggesting most of the patients around 30 years. Those studies showed a male-to-female rate of 1:3. The follow-up of just 34 patients was described (3-4,7,14), and six months was the minimum period.

**Table 2:**
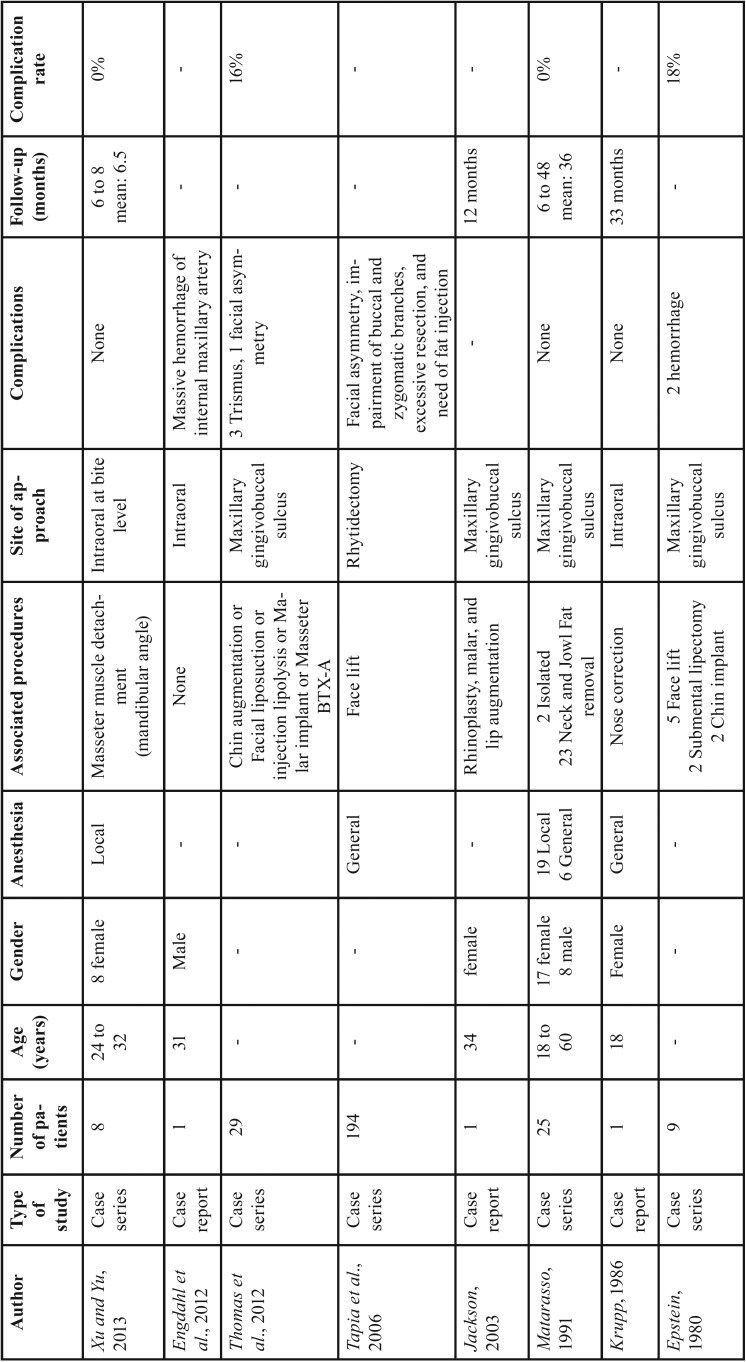
Descriptive analysis of included studies in final review.

The anesthesia regimen was defined according to the type of associated procedure. The isolate removal of BFP can be performed under local anesthesia, however when the extensive associated procedures are performed (neck and jowl fat removal, nose correction, and face lift) the general anesthesia was chosen. None of studies described the use of preoperative image exam to determine BFP extension and volume.

Regarding the complications, there was a report of extensive hemorrhage, facial nerve impairment, facial asymmetry, and trismus. Analyzing just the case series (3,6-7,10), 71 patients undergone to BFP removal and just six (8.45%) presented complications as trismus, facial asymmetry and hemorrhage.

## Discussion

The partial removal of BFP or “partial buccal lipectomy” aims to sculpt the lower face and reduce rounded faces. The procedure is related to the concept of an “inverted triangle of youth” that may increase the beauty. This concept is defined by an angular facial appearance resulted from a leaner face with a high malar region (3,6). In 1980, Epstein (10) first reported the BFP removal to improve the facial aesthetics. Although it is not a novel procedure, nowadays there is an extensive commercial marketing with appeal to facial aesthetics (6), and the procedure is disseminated as a routine. Thus, this systematic literature review aimed to identify the current state of BFP removal and the possible effects.

In the reviewed literature, the procedure can be indicated for cases with rounded faces or with presence of BFP pseudoherniation (2-4,8,10). When pseudoherniation is diagnosed, the patient shows a small rounded contour irregularity in the cheek due to weakening of BFP fascia (8). Patients with rounded faces show cheek/midface fullness despite appropriate weight for height (3,10). In both cases, the procedure’s goal is to reduce midface fullness, highlight the zygomatic prominence and the mandibular body, and remove any soft tissue asymmetry (3,10,14). Only one absolute contraindication was found in the literature, the procedure is contraindicated for patients with hemifacial atrophy, where BFP atrophy is a well-known component (1).

Another possible indication is as adjunct procedure in facial feminization surgery, aiming to change the characteristics of a male face to a female one. The female face usually has a triangular shape, with the base of an inverted triangle in a line drawn between the maximum prominence of each zygoma and the apex to the chin (16). Thus, as reported, the BFP removal may enhance those aspects and outcomes.

Concerning the long-term effects and facial aging, none of the included studies evaluated those features. Krupp (4) (1986) theorized that a severe weight loss associated with BFP removal could result in deep hollows in the cheek, however this situation was not found in the included studies. Matarasso (3) (1991) reports that there is a weak relationship between corporeal fat and BFP size, and even with aging and the characteristic loss of fat, the BFP remain in a relatively fixed size, demonstrating BFP resistance to lipolysis (2,4). Thus, patients with excessive BFP size will maintain this volume trough aging, and its removal may result in a general aesthetic improvement through time. However, it is important to highlight that there is a lack of knowledge regarding to the long-term effects of the procedure and its role in the facial aging.

The maintenance of BFP size over time is confirmed by image studies. Generally, the volume of the BFP is constant in adults (8). Volumetric evaluations show that the BFP grows between childhood and adult life, increasing from 4000 mm3 to 8000 mm3, and between the 20 and 50 years’ declines to 7000 mm3 (9). Also, volumetric analysis demonstrate that BFP is not always symmetric, especially in post-trauma patients (14). Therefore, a preoperative MRI should be the chosen image exam to determine the extension and symmetry of BFP (8). It is interest to observe that any of the included studies reported on the use of preoperative image exams for surgical planning. Thus, would be desirable to future studies the preoperative imagining evaluation in order to define the real necessity of those exams.

Regarding the selection of the surgical technique, there are two approaches to BFP removal: associated with facelift procedure (rhythidectomy) or by intraoral incision. When associated with rhythidectomy, it is expected impairment of buccal and zygomatic branches of the facial nerve (2). Thus, the safer method is to approach the BFP through intraoral incision (3,8,10,14). This incision can be performed at bite level or in maxillary gingivobuccal sulcus. The main difference between these incisions is the relationship with parotid duct, however no difference was observed in the studies regarding to complication rates or procedure’s difficulty. Xu and Yu (7) (2013) demonstrated a case series of BFP removal concomitant to masseter muscle detachment, which the incision at bite level seems more indicated. Nonetheless, there is no comparative study between those techniques, so the indications, damage to adjacent structures and postoperative aspects should be evaluated by future clinical trials.

The complication rate of the included studies, considering the reported results, amounts to 8.45% of the treated patients. This list included hemorrhage, facial asymmetry, and trismus. Although the reported complications are considered minor, injuries to parotid duct and facial nerve may occur (3-4,10,14). Engdahl, *et al.* (15) (2012), reported a massive hemorrhage of internal maxillary artery after intraoral BFP removal, in which the patient almost died. The lack of information about complications suggests that prospective clinical trials should be performed in order to define the potential complications of the technique.

It is important to highlight the differences between intraoral approach and face lift procedure. Besides the anesthesia regimen, the surgical anatomy is completely different. Most of complications are related to chosen approach and not to BFP removal itself. The face lift presented major complications as impairment of buccal and zygomatic branches (2). Those complications occurred due to damage to structures involved in the facial approach (3). The most important structure related with intraoral approach is the parotid duct. As reported, to avoid damage to this structure, the incision is preconized above (maxillary gingivobuccal sulcus) or below (at bite level) of the duct.

Although BFP removal may be performed isolated, a variety of associated procedures were found in this systematic review, including face lift, submental lipoplasty, rhinoplasty, malar, and chin implants, lip augmentation, masseter detachment and Botulinum toxin (BTX-A) injection (2-4,6-7,10,14). This high number of procedures occurred due to the aesthetic purpose of BFP removal. Usually, those patients seek not only for rounded face correction but also for others plastic procedures (6). Regarding to anesthesia regimen, both local and general were observed. Generally, the intraoral BFP removal is performed under local anesthesia (3,10), however the presence of concomitant procedures may indicate general anesthesia.

It is important to notice that none of all included articles was a clinical trial, hence all had a high risk of bias according to PRISMA evaluation. This fact shows a limitation of this systematic review, because there is a lack of clinical studies about BFP removal and its effects. This information shows the need of randomized clinical trials to compare the different methods of technique, to evaluate long-term effects in facial aging and function and to report complication types and rates.

In conclusion, all studies reported that BFP removal has an initial favorable outcome regarding facial aesthetics. The presented complication rate was low, without severe damages reported. However, the need of preoperative image exam, long-term effects in facial aging, and difference between intraoral techniques are not clear. Moreover, the amount of removal is not described and if it is excessive may result in an unfavorable outcome.
